# Drug-Induced Liver Injury during Consolidation Therapy in Childhood Acute Lymphoblastic Leukemia as Assessed for Causality Using the Updated RUCAM

**DOI:** 10.1155/2022/5914593

**Published:** 2022-03-24

**Authors:** Fu-Li Qin, Guo-You Sang, Xiao-Qin Zou, Dao-Hai Cheng

**Affiliations:** ^1^Department of Pharmacy, First Affiliated Hospital of Guangxi Medical University, Nanning, China; ^2^Department of Pharmacy, Guangxi Zhuang Autonomous Region Workers' Hospital, Nanning, China; ^3^Department of Medical Research, First Affiliated Hospital of Guangxi Medical University, Nanning, China

## Abstract

**Purpose:**

The presence of serious toxicities is a major problem in the treatment of childhood acute lymphoblastic leukemia (ALL). The objective of this research is to evaluate drug-induced liver injury (DILI) during consolidation therapy in childhood ALL.

**Methods:**

Clinical data of pediatric patients who received consolidation therapy between August 2012 and July 2018 were collected. Characteristics (incidences and patterns) of DILI at different stratifications were determined. Risks of DILI were evaluated using binary logistic regression analysis. Drug causality assessment was carried out by the updated Roussel Uclaf Causality Assessment Method (RUCAM).

**Results:**

Patients with high risk (HR) and standard risk (SR)/intermediate risk (IR) received 270 and 1539 courses of consolidation therapy, respectively; among these courses, 15 (5.6%) and 38 (2.5%) developed DILI. The occurrences of DILI in SR/IR patients were primarily associated with age (≤5.2 years), treatment course (≥5), and baseline serum parameters before treatment (cystatin C > 0.79 mg/L, albumin ≤45 g/L, and gamma-glutamyl transpeptidase (GGT) > 17 U/L). The ROC curve generated using the parameters assigned to specific values achieved an area under the curve (AUC) of 0.846 (95% CI 0.827–0.863) with a cutoff value of 3, and the sensitivity and specificity were 94.7% and 62.3%, respectively. For HR patients, a decrease in baseline albumin and elevation of baseline liver enzymes (GGT and aspartate aminotransferase) were observed in DILI cases compared with the non-DILI subjects. In the SR/IR group with DILI, the causality gradings for high-dose methotrexate (HD-MTX) were highly probable in 5 (13.2%) cases, probable in 31 (81.6%) cases, and possible in 2 (5.3%) cases. Among the DILI cases in HR-1, HR-2, and HR-3 groups, high causality gradings (probable + highly probable) were detected in “100% of HD-MTX + 57% of high-dose cytarabine (HD-Ara-C),” “100% of HD-MTX + 20% of pegylated asparaginase (PEG-ASP),” and “100% of HD-Ara-C + 33.3% of PEG-ASP,” respectively.

**Conclusion:**

Incidence of DILI in HR patients was significantly higher than that in SR/IR patients. A number of potential risk factors were identified, among which the preexisting liver conditions were suggested as shared risk factors in all stratification groups. HD-MTX, HD-Ara-C, and PEG-ASP were the main causative agents of DILI. The knowledge generated from this study will be helpful for understanding characteristics of DILI during consolidation treatment in childhood ALL.

## 1. Introduction

Acute lymphoblastic leukemia (ALL) is the most common malignancy in children [1]. The treatment of childhood ALL usually consists of following phases: induction, early intensification, consolidation, delayed intensification, and maintenance [2]. During the consolidation phase, patients often receive multiple chemotherapeutic agents, such as 6-mercaptopurine (6-MP), methotrexate (MTX), asparaginase (ASP), cyclophosphamide (CTX), etoposide (VP-16), and cytarabine (Ara-C). These chemotherapeutic drugs have a variety of adverse effects, especially when used at high doses or administered in combination. As a result, toxicities associated with treatment are the main obstacles in consolidation therapy of childhood ALL [2, 3].

The development of severe toxicities secondary to chemotherapy not only increases the cost of treatment but also delays scheduled therapy and jeopardizes the cure rate for these patients [4]. Among those toxicities, hepatotoxicity is one of the most important barriers during ALL chemotherapy. However, to our knowledge, overall hepatotoxicity during consolidation therapy, especially high-dose MTX (HD-MTX)-based polychemotherapy, has not been fully elucidated in Chinese pediatric patients. In the present study, we characterized the incidences, types, risk factors, and causality assessment of drug-induced liver injury (DILI) during consolidation therapy in childhood ALL.

## 2. Patients and Methods

### 2.1. Study Design

**Figure 1:**
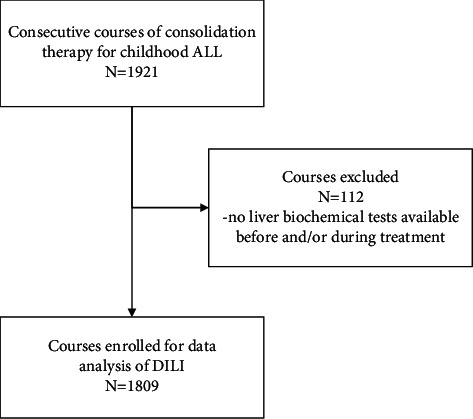
Flow chart of courses inclusion. ALL, acute lymphoblastic leukemia; DILI, drug-induced liver injury.

We retrospectively collected data of pediatric ALL patients who received consolidation therapy at the First Affiliated Hospital of Guangxi Medical University from August 2012 to July 2018. In total, there were 1921 consecutive courses of consolidation therapy during the study period. After excluding those with incomplete data, 1809 courses were included in the analysis (Figure 1). Incidences and patterns of DILI were determined. Risks at various stratifications were evaluated, and drug causality assessment was performed. Ethical approval was obtained from the Ethical Review Committee of the First Affiliated Hospital of Guangxi Medical University (2021(KY-E-111)).

### 2.2. Patients

Risk stratification (standard risk, SR; intermediate risk, IR; and high risk, HR) of patients were defined based on their clinical features (e.g., age, initial leukocytosis, immunophenotype, and cytogenetics) and early prednisone treatment response [5]. The treatment of consolidation was conducted at about 2 weeks after the end of early intensification chemotherapy according to the Chinese Children Leukemia Group (CCLG) 2008 protocol [5].

### 2.3. Treatments

For SR and IR patients, they received 2 000 and 5 000 mg per meter squared (mg/m^2^) body surface area (BSA) of HD-MTX as a 24-hour infusion, respectively. This process was repeated 4∼6 times with an interval of at least 14 days. 25 mg/m^2^/d of 6-MP was taken orally on empty stomach before bedtime. A single intrathecal injection of MTX for SR patients and triple intrathecal therapy (TIT) composed of MTX, Ara-C, and dexamethasone (DXM) for IR patients were administered at 2 hours after HD-MTX infusion. 15 mg/m^2^ of leucovorin (LV) was given at 42 h after the start of MTX infusion, and the dosage of LV at 48 h and later (every 6 h) were adjusted based on MTX plasma concentration.

For HR patients, they were treated with HR-1, HR-2, and HR-3 schemes successively. (1) HR-1: DXM 20 mg/m^2^/d, administered either orally or intravenously (IV), d1–5; Vincristine (VCR) 1.5 mg/m^2^/d, IV, d1 and d6; HD-MTX 5 000 mg/m^2^/d, administered IV as a 24-hour infusion, d1; CTX 200 mg/m^2^ every 12 hours, IV over 1 hour, totally 5 times on d2–4; High-dose Ara-C (HD-Ara-C) 2 000 mg/m^2^ every 12 hours, IV over 3 hours, totally twice on d5; Pegylated ASP (PEG-ASP) 2 500 U/m^2^/d, intramuscularly (IM), d6. TIT was administered on d1. (2) HR-2: Regimens of DXM, HD-MTX, PEG-ASP and TIT were the same as those in HR-1; Vindesine (VDS) 3 mg/m^2^/d, IV slowly, d1 and d6; Ifosfamide (IFO) 800 mg/m^2^ every 12 hours, IV over 1 hour, totally 5 times on d2–4. Daunorubicin (DNR) 30 mg/m^2^/d, IV, d5. (3) HR-3: Regimens of DXM and PEG-ASP were the same as those in HR-1. HD-Ara-C 2 000 mg/m^2^ every 12 hours, IV over 3 hours, totally 4 times on d1–2. VP-16 100 mg/m^2^ every 12 hours, IV over 1 hour, totally 5 times on d3–5. TIT was administered on d5. The intervals between schemes were about 2 weeks.The sequential treatment with the HR-1, HR-2, and HR-3 schemes was repeated once. The drug dosages of these schemes are summarized in Table 1.

### 2.4. Data Collection

We retrospectively collected information from the hospital information system (HIS). Parameter selection criteria for developing models were based on the resources available from the HIS and existing literature. These parameters include patients' demographic features (e.g., age, sex, ethnicity, body mass index (BMI), BSA, and courses), biochemical parameters (e.g., renal function, liver function, and blood routine), and medication information (e.g., drug doses, administration of potential hepatotoxic agents, and prophylactic use of hepatoprotective drugs). The laboratory tests for liver function were performed as baseline measurements before each course of chemotherapy and were conducted at least once during treatment based on the patients' status. The median detection frequency of liver function after initiating chemotherapy was 1 (range, 1–13). The median detection time of liver function was 3 days (range, 1–25 days) after the start of chemotherapy.

### 2.5. Definition and Pattern of DILI

DILI was detected by serum biochemical criteria, which include alanine aminotransferase (ALT), alkaline phosphatase (ALP), and bilirubin [6]. Here, subjects were defined as having DILI if they met one of the following criteria: (1) ALT ≥5 × upper limit of normal (ULN), (2) ALP ≥2 × ULN, or (3) ALT ≥3 × ULN and simultaneous bilirubin >2 × ULN. If the liver biochemistry was abnormally elevated before consolidation therapy, ULN was replaced by the baseline value. The pattern of DILI was identified using the *R* value, where *R* = (ALT/ULN)/(ALP/ULN). DILI was considered “hepatocellular” when *R* ≥ 5, “cholestatic” when *R* ≤ 2, and “mixed” when 2 < *R* < 5. The ULN of ALT was 60 U/L for boys and 45 U/L for girls. The ULN of total bilirubin was 20.5 *μ*mol/L. The ULN of ALP was 500 U/L (age <2 years) and 750 U/L (2–14 years). For children aged >14 years, the ULN of ALP was 125 U/L for boys and 100 U/L for girls.

### 2.6. Statistical Analysis

As the chemotherapy regimens in SR and IR patients were roughly similar, data of SR and IR groups were merged for statistical analysis. Continuous variables were presented as mean ± standard deviations (SD), and categorical data were expressed as numbers (percentages). The SPSS 20.0 software (SPSS Inc., Chicago, IL, USA) was used for the statistical analysis of the data. For the SR/IR patients, univariate analysis was conducted first and variables with a *p* value of less than 0.20 were included in binary logistic regression analysis using a Forward LR method. Cutoff values of the identified independent risk factors were determined by the receiver operating characteristic (ROC) curve. The independent factors were assigned values of 0, 1, and 2 based on the rounded regression coefficient (B) and their role in the development of DILI. The area under the curve (AUC), sensitivity, and specificity of the final model were assessed using the ROC curve. For the HR patients, only univariate analysis was performed due to the limited number of DILI cases. The updated Roussel Uclaf Causality Assessment Method (RUCAM) was used to determine the causal relationship between liver injury and suspected drugs [6].

## 3. Results

### 3.1. Characteristics of DILI

A total of 1809 eligible courses involving 463 patients, 293 boys and 170 girls, were enrolled for analysis in the present study. There were 49 patients in the HR group and 414 patients in the SR/IR groups who underwent 270 and 1539 courses of consolidation therapy, respectively. The median age at consolidation therapy was 6.4 years (range, 1.1–15.7 years). The patients were treated with a median number of 3 courses (range, 1–9 courses). The median time to first detection of DILI after the start of chemotherapy was 3 days (range, 3–8 days). The incidences of DILI in HR-1 and total HR (HR-1 + HR-2 + HR-3)courses were 7.4% (7/94) and 5.6% (15/270), respectively, which were both significantly higher (*p* < 0.05) than that in the SR/IR group (2.5%, 38/1539). All DILI cases presented with a hepatocellular pattern. Compared with non-DILI patients, DILI subjects had longer hospital stays and greater hospital expenses in the SR/IR population (both *p* < 0.05) (Table 2).

### 3.2. Risk Factors of DILI in SR/IR Patients

**Figure 2:**
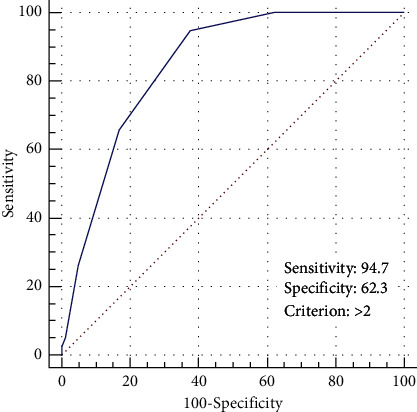
ROC analysis of combined risk factors for predicting DILI in SR/IR cases.

The demographic and laboratory characteristics of SR/IR patients and univariate analysis are shown in Table 3. The occurrences of DILI in SR/IR patients were primarily associated with age (≤5.2 years), treatment course (≥5), and baseline serum parameters before treatment (cystatin C > 0.79 mg/L, albumin ≤45 g/L, and GGT >17 U/L) (Table 4). The treatment course and baseline GGT were assigned a value of 2, and the remaining factors were denoted as 1 if they reached the above threshold values, otherwise they were assigned as 0. The area under the ROC curve was 0.846 (95% CI 0.827–0.863) with a cutoff value of 3, and the sensitivity and specificity were 94.7% and 62.3%, respectively (Figure 2).

### 3.3. Risk Factors of DILI in HR Patients

The descriptive statistics of HR patients and univariate analysis are shown in Table 5. The baseline albumin levels were significantly lower (*p* < 0.05) in the DILI group than in the non-DILI group, whereas GGT and AST were significantly higher (both *p* < 0.05).

### 3.4. Drug Causality Assessment Using the Updated RUCAM

As all cases with liver injury presented with a hepatocellular type, the RUCAM worksheet for hepatocellular injury was undertaken to assess the strength of association between drug exposure and liver injury [6]. For each patient with suspected DILI, based on the defined criteria, the score of each item was assigned, the sum of which provided a final score with causality grading that reflects the likelihood that the hepatic injury is due to a specific medication: ≥9, highly probable; 6∼8, probable; 3∼5, possible; 1∼2, unlikely; and ≤0, excluded.

In the SR/IR group with DILI, the high causality (probable + highly probable) relationship with HD-MTX was found in 95% (36/38) cases (Table 6). Among the DILI cases in HR groups, 100% (7/7) of HD-MTX and 57% (4/7) of HD-Ara-C in HR-1, 100% (5/5) of HD-MTX and 20% (1/5) of PEG-ASP in HR-2, and 100% (3/3) of HD-Ara-C and 33.3% (1/3) of PEG-ASP in HR-3 were rated as high causality degrees (probable + highly probable) by the updated RUCAM (Table 7). For other drugs (e.g., DXM, VCR, CTX, VDS, IFO, DNR, and VP-16), they were scored as a possible, unlikely, or excluded cause of DILI (Table 6). There were 3 cases of HD-Ara-C and 13 cases of PEG-ASP exposure initiated on or after the onset of liver injury, and they were therefore classified as unrelated to DILI.

## 4. Discussion

The cause of DILI is not completely understood but potentially involves multiple host demographic, clinical, and laboratory features [7]. However, the intricate interplay between these features may yield different conclusions concerning DILI risk factors in diverse research contexts [8]. As for childhood ALL patients, the clinical conditions of the patients, existing comorbidities, and polypharmacy make the assessment particularly challenging. In this study, we evaluated these factors at the DILI onset during consolidation therapy in childhood. HD-MTX treatment and preexisting liver conditions were considered as common underlying risk factors in patients receiving SR, IR, HR-1, and HR-2 regimens.

Indeed, it is generally considered that elevations of serum aminotransferases after HD-MTX are transient and reversible and do not lead to chronicity of liver disease [9]. However, we found that DILI subjects in the SR/IR group had longer hospital stays and greater hospital expenses than the non-DILI patients, although no significant differences were observed in HR subgroups, indicating that the development of DILI may increase the time cost and economic burden of leukemia treatment. This shows that it is warranted to study DILI during consolidation therapy in childhood ALL.

The overall incidence of DILI in HR patients (5.6%, 15/270) was significantly higher (*p* < 0.05) than that in SR/IR patients (2.5%, 38/1539), which was paralleled by increased exposure to drugs, implying that the more chemotherapy agents the patients received, the higher incidence of DILI occurred. This result is in line with what has been reported in the literature [10]. On the other hand, the incidence of DILI in IR patients who received 5 g/m^2^ of HD-MTX monotherapy was found to be 2.9% (25/871) and was significantly lower (*p* < 0.05) than that in the HR-1 group (7.4%, 7/94). However, no statistically significant difference (*p* > 0.05) was observed in the incidence of DILI between HR-2 (5.5%, 5/91) and IR groups (2.9%, 25/871). These results indicate that, apart from HD-MTX, the concomitant drugs may also play a contributing role in the development of DILI in HR patients.

Causality is defined as the causal relationship between medication use and adverse drug reaction (ADR). The updated RUCAM, a validated causality assessment method, is the most commonly used tool worldwide for assessing causality in suspected DILI cases [11]. Concomitant/sequential exposure to multiple drugs is a prominent feature during consolidation therapy for HR patients. Therefore, it would be essential to identify which drug is most likely to cause liver injury in this category of patients. In this study, we performed separate causality assessment for each implicated drug with the updated RUCAM. To assess the strength of association between liver injury and the suspected drugs, we particularly focused on the high causality gradings of “probable” (score 6∼8) or “highly probable” (score ≥9) likelihood scores. Within the DILI cases for evaluation, HD-MTX, HD-Ara-C, and PEG-ASP were identified as having higher RUCAM scores than other concomitant drugs. In general, 100% (12/12) of HD-MTX in “HR-1 + HR-2”, 70% (7/10) of HD-Ara-C in “HR-1 + HR-3,” and 13% (2/15) of PEG-ASP in “HR-1 + HR-2 + HR-3” were assessed as high-degree causality (probable or highly probable). It can be concluded that HD-MTX, HD-Ara-C, and PEG-ASP are the most likely causative agents of DILI in HR patients. In this study, as HR-1, HR-2, and HR-3 patients underwent combination therapy regimens containing “HD-MTX + HD-Ara-C,” “HD-MTX + PEG-ASP,” and “HD-Ara-C + PEG-ASP,” respectively, this might partially explain why HR patients had a higher incidence of DILI than the SR/IR subjects who received single HD-MTX monotherapy.

It is worth noting that exclusion of alternative causes is a crucial element of causality assessment in a suspected DILI. In this study, by using the updated RUCAM methodology (element: search for alternative causes), we carefully assessed the non-drug-related etiologies (such as alcohol, viral infections, and others) that could lead to a similar pattern of liver injury. All these potential alternative causes were reasonably ruled out for our patients after thorough investigation.

In host-related factors, the most significant factor identified in our study was the presence of raised liver enzymes before chemotherapy, which coincides with previous reports [12]. In this situation, patients' liver function tends to aggravate when challenged with specific chemotherapy drugs. Regarding the age, our results showed that, for SR and IR patients, younger children (≤5.2 years) might have a higher risk of DILI induced by HD-MTX. Previous study has shown that female patients appear to be more susceptible than male patients to DILI following exposure to certain therapeutic drugs, and a number of hypotheses have been put forward to explain the sex bias in susceptibility to DILI, including pharmacokinetics or pharmacodynamics, specific hormonal effects, and differences in the response of the immune system to drugs [13]. However, differences in male and female incidence rates were not observed in SR/IR (23/981 vs. 15/558, *p* > 0.05) or HR groups (6/171 vs. 9/99, *p* > 0.05) in this study. Similar to our results, a US Drug-Induced Liver Injury Network (DILIN) prospective study failed to show apparent correlation between female sex and severity of DILI [14]. This discrepancy indicates that the effect of gender on DILI incidence may vary depending on the clinical contexts such as study designs, interventions, patient populations, and regions. In addition, the components of metabolic syndrome (e.g., obesity and diabetes mellitus) are considered risk factors for drug-associated fatty liver disease (DAFLD) in patients treated with MTX [8]. However, in this study, we did not observe significant difference in BMI between the DILI group and the non-DILI group, possibly due to the different clinical conditions in the present study and those reported by other investigators [15, 16].

Patients of SR/IR receiving five or more courses of therapy exhibited a higher risk of DILI, which highlights that more attention should be paid as the dosing frequency and cumulative dose of MTX increase. Our data also showed that baseline cystatin C had a positive association with DILI, which has rarely been reported in previous studies.

Previous studies have shown that adjuvants like folic acid and vitamin E can reduce the incidence of MTX hepatotoxicity in rheumatoid arthritis [17–19]. However, whether hepatoprotective drugs (e.g., reduced glutathione, glycyrrhizin, and glucurolactone) reduce the occurrence of DILI during consolidation chemotherapy for childhood ALL remains largely unclear. At present, there are no available data concerning their effects on the efficacy of chemotherapy, particularly for regimens containing HD-MTX. In addition, the potential interaction between hepatoprotective drugs and chemotherapy agents may increase the complexity of metabolism of the latter. Therefore, whether the prophylactic use of hepatoprotective drugs should be recommended and routinely administered before chemotherapy in ALL clinical practice remains questionable. In our study, patients receiving these hepatoprotective drugs did not exhibit reduced DILI, indicating that such agents may not be required before consolidation therapy.

This study has a few limitations that need to be considered. First, due to the retrospective nature of this study, missing data are a well-known source of bias in research. For example, in this study, two cases of DILI in the SR/IR group were assessed as “possible” causality because their biochemical tests were not performed at certain time points after the treatment, which would decrease RUCAM scores thereby leading to underestimation in the causality assessment. Second, it should be noted that the role of other potential risk factors cannot be excluded. As is known, inheritance or genetic variations are crucial contributors to DILI [20, 21]. For instance, human leukocyte antigen (HLA) alleles have been suggested as strong risk factors for the development of DILI with a range of drugs [22]. Methylenetetrahydrofolate reductase (MTHFR) C677T gene polymorphisms have also been associated with hepatotoxicity due to MTX [8]. However, the effect of such genetic markers on DILI was not addressed in this study. Third, limited by the resources currently available, the number of courses recruited for HR patients was relatively limited. Further studies with larger case numbers need to be conducted for a better understanding of DILI in HR patients. Fourth, apart from liver biochemical blood tests, recent evidence has highlighted the importance of ultrasound evaluation and measurement of hepatic stiffness in ALL patients treated with hepatotoxic drugs. These tools are important to support screening of other forms of liver-related adverse effects, including DAFLD in patients treated with MTX and portal hypertension-related complications (e.g., sinusoidal obstruction syndrome/veno-occlusive disease (SOS/VOD) and hepatomegaly) in patients treated with inotuzumab ozogamicin [8, 23]. The lack of these noninvasive assessments of the liver is another possible limitation of our study and needs to be studied in future work.

In conclusion, the occurrences of DILI in SR and IR patients, who received HD-MTX monotherapy, were primarily associated with age, treatment course, and baseline serum parameters before treatment (e.g., cystatin C, albumin, and GGT). As for HR patients, preexisting liver conditions prior to treatment were potential risk factors of DILI. HD-MTX, HD-Ara-C, and PEG-ASP were the most likely causative agents of DILI during consolidation therapy.

## Figures and Tables

**Table 1:** Primary chemotherapeutic agents in HR consolidation therapy.

Scheme	Agent	Daily dose	Duration (day)	Route	*d*1	*d*2	*d*3	*d*4	*d*5	*d*6
*HR-1*	DXM	20 mg/m^2^	5	OR/IV	√	√	√	√	√	
	VCR	1.5 mg/m^2^	2	IV	√					√
	HD-MTX	5 000 mg/m^2^	1	IV	√					
	CTX	400 mg/m^2^	2.5	IV		√	√	√		
	HD-Ara-C	4 000 mg/m^2^	1	IV					√	
	PEG-ASP	2 500 U/m^2^	1	IM						√
	TIT	Based on age	1	IT	√					

*HR-2*	DXM	20 mg/m^2^	5	OR/IV	√	√	√	√	√	
	VDS	3 mg/m^2^	2	IV	√					√
	HD-MTX	5000 mg/m^2^	1	IV	√					
	IFO	1 600 mg/m^2^	2.5	IV		√	√	√		
	DNR	30 mg/m^2^	1	IV					√	
	PEG-ASP	2500 U/m^2^	1	IM						√
	TIT	Based on age	1	IT	√					

*HR-3*	DXM	20 mg/m^2^	5	OR/IV	√	√	√	√	√	
	HD-Ara-C	4000 mg/m^2^	2	IV	√	√				
	VP-16	200 mg/m^2^	2.5	IV			√	√	√	
	PEG-ASP	2500 U/m^2^	1	IM						√
	TIT	Based on age	1	IT					√	

Note: HR, high risk; DXM, dexamethasone; VCR, vincristine; HD-MTX, high-dose methotrexate; CTX, cyclophosphamide; HD-Ara-C, high-dose cytarabine; PEG-ASP, pegylated asparaginase; TIT, triple intrathecal therapy; VDS, vindesine; IFO, ifosfamide; DNR, daunorubicin; VP-16, etoposide; OR, orally; IV, intravenously; IM, intramuscularly; IT, intrathecally.

**Table 2:** Characteristics of DILI in different regimens.

	SR/IR (*n* = 1539)	HR-1 (*n* = 94)	HR-2 (*n* = 91)	HR-3 (*n* = 85)
DILI, *n* (%)	38 (2.5)	7 (7.4)^a^	5 (5.5)	3 (3.5)
Hepatocellular pattern of DILI, *n* (%)	38 (100)	7 (100)	5 (100)	3 (100)
*Hospital stay, days*				
DILI	8.2 ± 4.4	12.1 ± 8.2	9.0 ± 5.2	6.3 ± 0.5
Non-DILI	4.9 ± 2.2^b^	7.8 ± 3.6	7.4 ± 3.2	6.8 ± 3.1
*Hospital expense, CNY*				
DILI	6868 ± 3125	25850 ± 21654	15019 ± 6749	10606 ± 837
Non-DILI	5051 ± 2532^b^	15967 ± 9808	13469 ± 9823	12688 ± 6337

SR/IR, standard risk/intermediate risk; HR, high risk; DILI, drug-induced liver injury; CNY, China Yuan. Superscripts “a” and “b” represent significant differences (*p* < 0.05) compared with SR/IR and DILI, respectively.

**Table 3:** Demographic and laboratory characteristics of DILI and non-DILI in SR/IR cases.

	DILI (*n* = 38)	Non-DILI (*n* = 1501)	Statistics	*p* value
Age, years	5.4 ± 2.8	6.4 ± 3.6	−1.433^∆^	0.152
*Gender, n (%)*				
Male	23 (2.3)	958 (97.7)	0.174^#^	0.676
Female	15 (2.7)	543 (97.3)		
Han ethnicity, *n* (%)	21 (55.3)	856 (57.0)	0.290^#^	0.865
Treatment course ≥5, *n* (%)	12 (31.6)	117 (7.8)	24.291^#^	<0.001
BMI, kg/m^2^	15.9 ± 1.6	16.2 ± 2.1	−0.418^∆^	0.676
MTX dose per BSA, g/m^2^	4.3 ± 1.1	4.0 ± 1.3	−1.512^∆^	0.131
48 h MTX concentration, *μ*mol/L	0.62 ± 0.53	1.14 ± 3.72	−0.286^∆^	0.775
Prophylactic agents, *n* (%)	30 (78.9)	1019 (67.9)	2.089^#^	0.148
*Baseline renal function*				
Creatinine, mg/dl	0.33 ± 0.11	0.37 ± 0.13	−1.965^∆^	0.049
Cystatin C, mg/L	0.78 ± 0.16	0.73 ± 0.15	−1.768^∆^	0.077
Urea, mmol/L	4.0 ± 1.5	3.9 ± 1.4	−0.697^∆^	0.486
Uric acid, *μ*mol/L	225.5 ± 72.0	226.6 ± 82.5	−0.668^∆^	0.504
*Baseline liver function*				
Albumin, g/L	44.3 ± 3.7	46.6 ± 3.4	−4.074^∆^	<0.001
Total bilirubin, *μ*mol/L	7.4 ± 4.9	6.3 ± 3.7	−1.834^∆^	0.067
ALT, U/L	38.6 ± 24.9	25.7 ± 26.3	−5.540^∆^	<0.001
GGT, U/L	40.9 ± 64.4	22.5 ± 20.4	−3.443^∆^	0.001
AST, U/L	35.3 ± 15.7	28.3 ± 12.0	−3.827^∆^	<0.001
ALP, U/L	218.0 ± 78.2	202.1 ± 65.9	−1.071^∆^	0.284
*Baseline blood routine*				
White blood cell count, ×10^9^/L	5.7 ± 3.3	5.8 ± 3.8	−0.202^∆^	0.840
Red blood cell count, ×10^12^/L	3.8 ± 0.5	3.8 ± 0.6	−0.303^∆^	0.762
Platelet count, ×10^9^/L	260.7 ± 122.6	297.2 ± 131.2	−1.796^∆^	0.073
Hemoglobin, g/L	110.1 ± 14.1	107.3 ± 14.2	−1.361^∆^	0.174

^∆^ *Z* value, statistics of the Mann–Whitney *U* test; ^#^*χ*^2^ value, statistics of the chi-square test. DILI, drug-induced liver injury; SR/IR, standard risk/intermediate risk; BMI, body mass index; MTX, methotrexate; BSA, body surface area; AST, aspartate aminotransferase; ALT, alanine aminotransferase; GGT, gamma-glutamyl transpeptidase; ALP, alkaline phosphatase.

**Table 4:** Variables in the equation for prediction of DILI in SR/IR patients.

	B	S.E.	Wald	Exp (B)	95% CI for EXP (B)	Sig.	Score
Age ≤5.2 years	1.338	0.376	12.663	3.813	(1.824, 7.969)	0.000	1
Treatment course ≥5	1.609	0.386	17.336	4.996	(2.343, 10.653)	0.000	2
Baseline cystatin C > 0.79 mg/L	0.726	0.345	4.429	2.066	(1.051, 4.060)	0.035	1
Baseline albumin ≤45 g/L	1.220	0.346	12.407	3.387	(1.718, 6.678)	0.000	1
Baseline GGT >17 U/L	1.750	0.421	17.305	5.756	(2.523, 13.128)	0.000	2

DILI, drug-induced liver injury; SR/IR, standard risk/intermediate risk; GGT, gamma-glutamyl transpeptidase.

**Table 5:** Demographic and laboratory characteristics of DILI and non-DILI in HR cases.

	DILI (*n* = 15)	Non-DILI (*n* = 255)	Statistics	*p* value
Age, years	6.0 ± 3.6	7.1 ± 3.5	−0.997^∆^	0.319
*Gender, n (%)*				
Male	6 (3.5)	165 (96.5)	3.724^#^	0.054
Female	9 (9.1)	90 (90.9)		
Han ethnicity, *n* (%)	6 (40.0)	128 (50.2)	0.589^#^	0.443
BMI, kg/m^2^	16.7 ± 1.3	16.7 ± 1.9	−0.612^∆^	0.540
Prophylactic agents, *n* (%)	9 (60.0)	109 (42.7)	1.714^#^	0.190
*Baseline renal function*				
Creatinine, mg/dl	0.30 ± 0.10	0.36 ± 0.13	−1.881^∆^	0.060
Cystatin C, mg/L	0.57 ± 0.14	0.60 ± 0.15	−0.250^∆^	0.803
Urea, mmol/L	3.88 ± 1.87	4.40 ± 1.66	−0.914^∆^	0.361
Uric acid, *μ*mol/L	219.2 ± 64.3	256.5 ± 82.5	−1.349^∆^	0.177
*Baseline liver function*				
Albumin, g/L	38.9 ± 5.3	42.3 ± 4.8	−2.335^∆^	0.020
Total bilirubin, *μ*mol/L	5.46 ± 2.01	5.60 ± 3.11	−0.502^∆^	0.616
ALT, U/L	42.8 ± 22.3	39.1 ± 30.6	−1.459^∆^	0.145
GGT, U/L	48.3 ± 26.0	39.8 ± 46.4	−2.332^∆^	0.020
AST, U/L	46.1 ± 10.4	37.1 ± 21.3	−3.580	＜0.001
ALP, U/L	190.4 ± 37.6	176.5 ± 55.9	−1.491^∆^	0.136
*Baseline blood routine*				
White blood cell count, ×10^9^/L	5.79 ± 3.42	5.04 ± 3.22	−1.113^∆^	0.266
Red blood cell count, ×10^12^/L	3.52 ± 0.42	3.59 ± 0.61	−0.393^∆^	0.694
Platelet count, ×10^9^/L	281.6 ± 113.4	301.1 ± 122.3	−0.689^∆^	0.491
Hemoglobin, g/L	94.7 ± 11.3	99.5 ± 12.9	−1.381^∆^	0.167

^∆^ *Z* value, statistics of the Mann–Whitney *U* test; ^#^*χ*^2^ value, statistics of the chi-square test. DILI, drug-induced liver injury; HR, high risk; HD-MTX, high-dose methotrexate; BMI, body mass index; AST, aspartate aminotransferase; ALT, alanine aminotransferase; GGT, gamma-glutamyl transpeptidase; ALP, alkaline phosphatase.

**Table 6:** Distribution of final scores of suspected agents in DILI cases using the updated RUCAM [6].

	Total frequencies	Highly probable (score ≥9)	Probable (score 6∼8)	Possible (score 3∼5)	Unlikely (score 1∼2)	Excluded (score ≤0)
*SR/IR*						
HD-MTX	38	5 (13.2%)	31 (81.6%)	2 (5.3%)	0 (0.0%)	0 (0.0%)
*HR*						
DXM	15	0 (0.0%)	0 (0.0%)	3 (20.0%)	12 (80.0%)	0 (0.0%)
VCR	9	0 (0.0%)	0 (0.0%)	9 (100.0%)	0 (0.0%)	0 (0.0%)
HD-MTX	12	0 (0.0%)	12 (100.0%)	0 (0.0%)	0 (0.0%)	0 (0.0%)
CTX	7	0 (0.0%)	0 (0.0%)	7 (100.0%)	0 (0.0%)	0 (0.0%)
HD-Ara-C	10	1 (10.0%)	6 (60.0%)	0 (0.0%)	0 (0.0%)	3 (30.0%)
PEG-ASP	15	0 (0.0%)	2 (13.3%)	0 (0.0%)	0 (0.0%)	13 (86.7%)
VDS	3	0 (0.0%)	0 (0.0%)	3 (100.0%)	0 (0.0%)	0 (0.0%)
IFO	5	0 (0.0%)	0 (0.0%)	5 (100.0%)	0 (0.0%)	0 (0.0%)
DNR	5	0 (0.0%)	0 (0.0%)	3 (60.0%)	0 (0.0%)	2 (40.0%)
VP-16	3	0 (0.0%)	0 (0.0%)	2 (66.7%)	0 (0.0%)	1 (33.3%)

RUCAM, Roussel Uclaf Causality Assessment Method; SR/IR, standard risk/intermediate risk; HR, high risk; HD-MTX, high-dose methotrexate; DXM, dexamethasone; VCR, Vincristine; CTX, cyclophosphamide; HD-Ara-C, high-dose cytarabine; PEG-ASP, pegylated asparaginase; VDS, vindesine; IFO, ifosfamide; DNR, daunorubicin; VP-16, etoposide.

**Table 7:** Cases assessed as high causality of “probable” or “highly probable” in HR groups.

	No. of cases	HD-MTX	HD-Ara-C	PEG-ASP
HR-1	7	7 (100.0%)	4 (57.0%)	0 (0.0%)
HR-2	5	5 (100.0%)	NA	1 (20.0%)
HR-3	3	NA	3 (100.0%)	1 (33.3%)

HR, high risk; HD-MTX, high-dose methotrexate; HD-Ara-C, high-dose cytarabine; PEG-ASP, pegylated asparaginase; NA, not applicable.

## Data Availability

The data used to support the findings of this study are included within the article.
